# Oncogenic functions of the EMT-related transcription factor ZEB1 in breast cancer

**DOI:** 10.1186/s12967-020-02240-z

**Published:** 2020-02-03

**Authors:** Hua-Tao Wu, Hui-Ting Zhong, Guan-Wu Li, Jia-Xin Shen, Qian-Qian Ye, Man-Li Zhang, Jing Liu

**Affiliations:** 1 Department of General Surgery, The First Affiliated Hospital of Shantou University Medical College, Shantou, 515041 China; 2 Changjiang Scholar’s Laboratory/Guangdong Provincial Key Laboratory for Diagnosis and Treatment of Breast Cancer, Shantou University Medical College, Shantou, 515041 China; 3 Open Laboratory for Tumor Molecular Biology, Department of Biochemistry, The Key Lab of Molecular Biology for High Cancer Incidence Coastal Chaoshan Area, Shantou University Medical College, Shantou, People’s Republic of China; 4 Department of Hematology, The First Affiliated Hospital of Shantou University Medical College, Shantou, People’s Republic of China; 5 Department of Physiology/Cancer Research Center, Shantou University Medical College, Shantou, 515041 China

**Keywords:** Zinc finger E-box binding homeobox 1 (ZEB1), Epithelial-mesenchymal transition (EMT), Breast cancer, Metastasis

## Abstract

Zinc finger E-box binding homeobox 1 (ZEB1, also termed TCF8 and δEF1) is a crucial member of the zinc finger-homeodomain transcription factor family, originally identified as a binding protein of the lens-specific δ1-crystalline enhancer and is a pivotal transcription factor in the epithelial-mesenchymal transition (EMT) process. ZEB1 also plays a vital role in embryonic development and cancer progression, including breast cancer progression. Increasing evidence suggests that ZEB1 stimulates tumor cells with mesenchymal traits and promotes multidrug resistance, proliferation, and metastasis, indicating the importance of ZEB1-induced EMT in cancer development. ZEB1 expression is regulated by multiple signaling pathways and components, including TGF-β, β-catenin, miRNA and other factors. Here, we summarize the recent discoveries of the functions and mechanisms of ZEB1 to understand the role of ZEB1 in EMT regulation in breast cancer.

## Background

Epithelial-mesenchymal transition (EMT) is the transdifferentiation process that causes epithelial cells to lose their epithelial characteristics, such as cell junctions and apical-basal polarity, and acquire mesenchymal characteristics, such as increased cell motility and invasive ability [1]. EMT is critical for normal developmental processes, such as embryonic stem cell differentiation and induced pluripotency, and is involved in disease development processes, such as wound healing, fibrosis, cancer stem cell (CSC) behaviors and cancer development/migration. EMT occurs in breast cancer, providing highly mobile and invasive cancer cells for further metastasis.

Over the past decades, extensive research has focused on the function of EMT and its underlying molecular mechanisms. Lamouille et al. reviewed the molecular mechanisms of EMT and summarized EMT transcription factors with their direct targets and signaling pathways [2]. Most of the EMT transcription factors regulated epithelial/mesenchymal markers (such as E-cadherin, N-cadherin, and vimentin) directly or indirectly during both normal development and physiopathological processes. Among these transcription factors, ZEB1 is a candidate repressor of E-cadherin [3] as are other zinc finger E-box binding (ZEB) family members [4], the Snail family of transcription factors [5] and basic helix-loop-helix (bHLH) factors [6].

The ZEB1 protein participates in the differentiation of multiple tissues, including bone tissue [7], smooth muscle tissue [8], neural tissue [9], etc. ZEB1 can decrease the expression of the epithelial marker E-cadherin [10] and related miR-200 s [11], resulting in the EMT process. Abnormal expression of ZEB1 has been reported in various human cancers, including pancreatic cancer [12], lung cancer [13], liver cancer [14], colon cancer [15], and breast cancer [16]. Additionally, the increased expression of ZEB1 enhanced the chemo/radioresistance of cancer cells [17], indicating that ZEB1 is not only involved in the oncogenesis and development of cancers but also affects the prognosis of cancer patients. This review will focus on the EMT transcription factor ZEB1 and its functions in the oncogenesis and development of breast cancer.

## Transcription factor ZEB1 and its structural characteristics

**Fig. 1:**
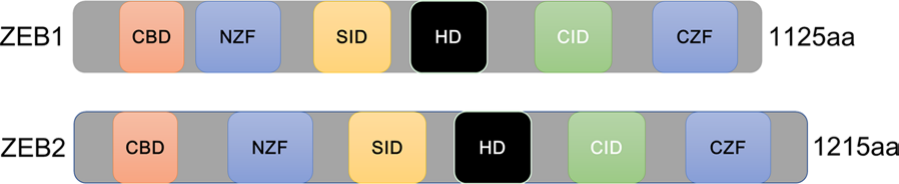
The schematic structure of ZEB1/2. The structures of ZEB1/2 are similar, with N-terminal and C-terminal zinc finger (NZF and CZF) and a central Homeodomain (HD). The ZEB1/2 protein interacted with other proteins through a corresponding binding domain, including the CAF/p300 binding domain (CBD) at the N-terminal, Smad interaction domain (SID) and CtBP interaction domain (CID) at the C-terminal

ZEB homeobox is a transcription factor family that includes ZEB1 (also known as TCF8 and δEF1) and ZEB2 (also known as SIP1) [18]. Both of them contain C_2_H_2_-type zinc fingers, the common DNA-binding motifs binding to paired CAGGTA/G E-box-like elements in the promoters of their target genes, and regulate cell differentiation and tissue-specific functions [19, 20]. *ZEB1* is located on Chr10p11.22 and encodes the 1117 amino acid ZEB1 protein, which mainly consists of the homologous structural domain homeodomain (HD) in the middle of the structure and the structures of the zinc finger N-terminal cluster (NZF) and C-terminal cluster (CZF) on both sides (Fig. 1).

## The expression pattern of ZEB1 in breast cancers and its molecular mechanism of transcriptional suppression

### The expression of ZEB1 in breast cancer

The expression level of ZEB1 is increased in triple-negative breast cancers (TNBCs) and basal-like breast cancers compared to the luminal subtype [21]. To understand the role of ZEB1 in TNBCs, Lehmann et al. compared the different gene expression levels between aggressive TNBC cancer cells (MDA-MB-231) with high ZEB1 levels and their corresponding ZEB1 knockdown cells, revealing that the expression of 60% of genes was upregulated after ZEB1 knockdown and that the remaining genes were downregulated [22]. They predicted potential direct or indirect target genes of the transcriptional repressor ZEB1 and suggested that abnormal expression of the gene set may be a predictor of poor survival, therapy resistance and increased metastatic risk in breast cancer [22].

### ZEB1 functions as a transcriptional suppressor

As mentioned before, the main transcriptional function of ZEB1 is suppressing the expression of its target genes, such as epithelial markers (E-cadherin), and correspondingly increasing the mesenchymal levels of vimentin and N-cadherin [23]. Eger et al. first reported ZEB1 as a direct transcriptional repressor of E-cadherin by physically binding to the proximal promoter of E-cadherin in breast cancers [10]. As a transcriptional repressor, it was identified that ZEB1 can also directly bind to the promoter of miR-190, resulting in transcriptional suppression of miR-190 expression, which can reverse the transforming growth factor (TGF)-β-induced EMT process [24]. Most importantly, the expression of the miR-200 family members was suppressed by ZEB1 binding to their promoters and was conversely involved in the regulation of ZEB1 levels as a reciprocal ZEB1/miR-200 feedback loop [25, 26].

A various set of cofactors were also recruited during the transcriptional suppression process of ZEB1 for its downstream target genes [27, 28], although only a few of them were reported [29]. ZEB1 activation requires interaction with PC2-CtBP-LSD1-LCoR or the yeast mating-type switching/sucrose non-fermenting (SWI/SNF) chromatin-remodeling protein BRG1 to form the ZEB1-Smad3-p300-P/CAF complex, affecting general transcription [28]. The effector of the Hippo/Yes-associated protein (YAP) pathway, YAP, can specifically and directly interact with ZEB1, converting ZEB1 from a transcriptional repressor to a transcriptional activator that binds to conserved TEAD-binding sites. As a result, functional cooperation between ZEB1 and YAP can stimulate the transcriptional activities of a ‘common ZEB1/YAP target gene set’, such as connective tissue growth factor (CTGF) and AXL receptor tyrosine kinase (AXL) [22].

Usually, the functional statuses of chromatin are identified by the covalent modification pattern of the N-terminal domains of the histones, indicating the transcriptional activity of their target genes. For example, histone H3 lysine 4 trimethylation (H3K4me3) was reported to be associated with transcriptional initiation [30], while lysine 79 dimethylation (H3K79me2) was associated with promoting transcriptional elongation [31]. Overall the combined effect of H3K4me3 and H3K79me2 contributes to the activation of gene transcription. In addition, lysine 27 trimethylation (H3K27me3) was suggested to contribute to transcriptional repression [32–35]. An innovative and interesting study found that luminal breast cancer cell lines exhibited only presence of H3K27me3 and the relative absence of 3K4me3 and H3K79me2 at the ZEB1 promoter [29]. Oppositely, in basal-like or basal CD44hi breast cancer cells, high expression levels of ZEB1 were controlled by H3K4me3 and H3K79me2 in its promoter, which did not have H3K27me3, indicating active transcription. More importantly, the ZEB1 promoter in basal CD44lo cells or plastic non-CSCs showed a bivalent chromatin configuration, enabling these cells to respond readily to microenvironmental signals, such as TGF-β [29].

### The regulation of ZEB1 expression and activity

As an important transcriptional factor, the expression of ZEB1 is also regulated at multiple levels [36]. Signal transducer and activator of transcription3 (STAT3) has been reported to induce the expression of ZEB1 [37]. Avtanski et al. identified that honokiol (HNK) is an active bisphenol molecule that inhibits the function of STAT3 by repressing STAT3 phosphorylation and the transactivation potential; STAT3 recruitment to the ZEB1 promoter is then reduced, which causes decreased ZEB1 expression and nuclear translocation. In addition, HNK-mediated STAT3 inactivation triggered the STAT3-mediated release of ZEB1 from the E-cadherin promoter, increasing E-cadherin expression and inhibiting EMT of breast cancer cells accordingly [37].

After the formation of the ZEB1 protein, insulin-like growth factor 1 (IGF-1) induced ZEB1 phosphorylation at residues T851, S852, and S853 by protein kinase C (PKC), showing involvement in the EMT process and resulting in transcriptional activity [38]. Another posttranslational modification of ZEB1 is polycomb protein (Pc2)-induced sumoylation at residues K347 and K777 [39].

The stability of EMT-related transcription factors is essential for initiating cellular EMT. Thus, the deubiquitinases (DUBs) were involved in counteracting polyubiquitination and proteasomal degradation of ZEB1 [40]; these DUBs include USP51, which upregulated ZEB1 and the mesenchymal markers by binding, deubiquitinating, and stabilizing ZEB1 [41]. In addition to DUBs, other factors, such as CSN5, an oncogene involved in various types of cancer, may also interrupt the degradation of ZEB1 by stabilizing ZEB1 by directly interacting with it [42]. The phosphorylation of ZEB1 by ataxia-telangiectasia mutated (ATM) kinase stabilizes ZEB1 in response to DNA damage [43].

The new findings related to posttranslational modifications that regulate ZEB1 provide an alternative pathway for precision medicine to treat breast cancer by targeting ZEB1.

## The reciprocal ZEB1/target feedback loops involved in breast cancers

As a transcriptional suppressor, ZEB1 is involved in many signaling pathways to inhibit the expression of its targets. Interestingly, some of the targets of ZEB1, such as the ZEB1/miR-200s loop, function as a regulator of ZEB1 to control the level and function of ZEB1.

### ZEB1/miR-200s loop

Independent investigations revealed that miR-200 family members, including miR-200a/b/c, miR-141 and miR-429, can revert the EMT process and are powerful inducers of epithelial differentiation. The mechanism of miR-200s suppressing ZEB1 involved posttranscriptional repression by binding to the 3′-untranslated region (UTR) of ZEB1 mRNA, which contains 8 miR-200s binding sites [11, 44, 45]. As mentioned before, in addition to the inhibitory function of miR-200s on ZEB1, the expression of miR-200s can be reversely suppressed by ZEB1 through direct binding to the highly conserved sites in the promoter common to the miR-200 family members [25]. Notably, ZEB1 and miR-200s have the opposite functions to regulate EMT and the characteristics of cancer cells but also reciprocally control the expression of each other, which is a double-negative feedback loop named the ZEB1/miR200 feedback loop [25, 26]. Depending on the different extracellular signals, the unstable status of the ZEB1/miR-200 feedback loop will strongly promote the expression and effect of one group and will correspondingly suppress those of the other group, resulting in the switch from one phenotype to another phenotype and stabilizing their epithelial/mesenchymal phenotype accordingly [46].

The ZEB1/miR-200 feedback loop was indicated to regulate EMT and, more importantly, was supposed to be a central switch for other crucial cellular functions, such as survival vs. apoptosis, stemness vs. differentiation, growth arrest vs. proliferation, and longevity vs. senescence [46]. In breast cancer, the studies of this loop mainly focused on mesenchymal-like characteristics involving different pathways or factors, such as the H-Ras signaling pathway [47], as well as the transcription factor zinc finger protein 217 (ZNF217) [25]. It was reported that autocrine TGF-β, which is upregulated by ZNF217, transcriptionally activated the expression of ZEB1 and finally exhibited feedback inhibition on miR-200c [48, 49]. Using the intertwined feedback loop, Bai et al. found the underlying mechanism of trastuzumab resistance and metastasis in breast cancer where miR-200c targeted ZNF217 and ZEB1 to suppress TGF-beta signaling [50].

### ZEB1/MYB loop

ZEB1/MYB is another reciprocal negative feedback circuit involved in the process of breast cancer development. Both ZEB1 and MYB are transcription factors that function as biological switches for molecular elements of their targets, affecting the tumor microenvironment and cell morphology. On one hand, ZEB1 transcriptionally suppressed the expression of MYB by controlling the positive feedback cycle of MYB. On the other hand, MYB was found to inhibit ZEB1 expression and correspondingly alleviate the ZEB1-mediated transcriptional repression of CDH1, restoring the epithelial phenotype [51]. The dual-negative regulation of ZEB1/MYC is thought to be the molecular mechanism of cell plasticity, regulating the state of tumor stem cells and promoting tumor invasion and metastasis.

### ZEB1/grainyhead-like-2 (GRHL2) loop

GRHL2 was found to be downregulated specifically in claudin-low subclass breast cancers and to suppress TGF-β-induced, Twist-induced or spontaneous EMT partially by suppressing ZEB1 expression by directly binding to the ZEB1 promoter [52]. Soon after, Cieply et al. revealed that (1) GRHL2 altered the Six1-DNA complex to inhibit the transactivation of the ZEB1 promoter; and that (2) the combination of TGF-β and Wnt activation interacted with ZEB1 directly, affecting the activities of the GRHL2 promoter to suppress its expression; these results indicate a reciprocal GRHL2 and ZEB1 feedback loop that controls epithelial/mesenchymal phenotypes and EMT progress [53].

### ZEB1/CD44s loop

In addition to negative feedback loops, ZEB1 was also involved in the positive dual promotion process with its targets. ZEB1 was correlated with maintaining stem cell specialties and cell survival in tumorigenesis [54], and a positive feedback loop between CD44s and ZEB1 was involved [46]. CD44s is the active type to promote the expression of ZEB1 and is controlled by epithelial splicing regulatory protein 1 (ESPR1) through alternative splicing of CD44, causing a shift from the variant CD44v to the standard CD44s isoform [55]. To maintain the stemness and mesenchymal features of cancer cells, ZEB1, in turn, inhibited transcription of the splicing factor ESRP1, causing the switch from CD44 splicing to preferentially express CD44s and resulting in self-sustained ZEB1/CD44s expression, which promoted both EMT and breast cancer development [56].

### ZEB1/hyaluronic acid synthase 2 (HAS2) loop

Hyaluronic acid (HA), synthesized mainly by HAS2, is one major extracellular matrix (ECM) proteoglycan that is enriched in mammary tumors [57]. Preca et al. reported a new positive feedback loop consisting of HAS2 and ZEB1 [58]. Extracellular HA contributed to ZEB1-driven EMT by triggering ZEB1 expression and was enhanced by HA-induced CD44s, indicating that HAS2 is necessary for TGF-β-induced EMT and that ZEB1 directly binds to the HAS2 promoter and activates its expression, further enhancing ZEB1 expression and shaping the microenvironment [58]. Interestingly, this positive ZEB1/HAS2/HA feedback loop will be facilitated by the ZEB1/ESRP1/CD44s loop, providing insight into a complex multifactorial positive feedback system.

## The oncogenic functions of ZEB1 in breast cancer and their underlying mechanisms

### Cell plasticity

CSCs, which are considered the subset of neoplastic cells in a highly tumorigenic state, contribute to phenotypic and functional heterogeneity in cancers [59]. Interestingly, an innovative study conducted by Chaffer et al. demonstrated that non-CSCs of human basal breast cancers are a plastic cell population, with the potential to switch from a non-CSC-state to a CSC-state; this switch is regulated by the condition of the ZEB1 promoter responding to microenvironmental signals (such as TGF-β) [29]. Accordingly, the active chromatin configuration of the ZEB1 promoter significantly increased the CD44lo/hi ratio, predicting the critical role of ZEB1 for the transition of cell types from CD44lo to CD44hi and for the generation of CSCs from non-CSC cells, maintaining the activities of CD44hi/CSC-like cells [29].

Similarly, ZEB1 was involved in Wnt/β-catenin signaling to promote cancer cell metastasis [60]. Aberrant levels of ZEB1 suppressed the expression of epithelial splicing regulatory proteins (ESRPs) [61, 62], promoting a shift from the epithelial isoform (CD44v8-9) to the mesenchymal isoform (CD44s) [63, 64]. Cyclin-dependent protein kinase-like 2 (CDKL2) was demonstrated to activate a positive feedback circuit, the ZEB1/E-cadherin/β-catenin signaling pathway, to enhance the mesenchymal characteristics and stem cell-like properties [65]. CDKL2 upregulated the expression of ZEB1 through increasing the promoter activities of ZEB1, while it has also been reported as a well-established transcriptional suppressor of E-cadherin [10]. Subsequently, a decreased level of E-cadherin destroyed the epithelial barrier [66], causing nuclear translocation of β-catenin and increased β-catenin/TCF4 transcriptional activity, which accordingly promoted ZEB1 promoter activity and transcriptional function [60], leading to further suppression of E-cadherin expression and sustaining activation of the positive feedback circuit. Finally, the EMT process was promoted by ZEB1 through the E-cadherin/β-catenin signaling pathway and strengthened the CD44 high mesenchymal properties under CDKL2 regulation [65].

Interestingly, in addition to the transition between the epithelial (E) and mesenchymal (M) states, Lu et al. proposed the “ternary chimera switch (TCS)” model and proposed a third state with intermediate levels of both miR-200 and ZEB, corresponding to the epithelial-mesenchymal (E/M) hybrid phenotype [67]. Further, Zhang et al. confirmed that the SNAIL1/miR-34 module forms a biswitch between the E and E/M transitions, and the ZEB1/miR-200 module is another biswitch for the transition from the E/M to M state [68].

On the other hand, Drosophila Lgl and its mammalian homologs, LLGL1/2, are scaffolding proteins that regulate the establishment of apical-basal polarity in normal epithelial cells, and LLGL2 also combined with SLC7A5 (a leucine transporter) and YKT6 (a regulator of membrane fusion) to form a trimeric complex, promoting leucine uptake, cell proliferation and resistance to endocrine treatment in ER-positive breast cancers [69]. ZEB1 has a specific property to modulate asymmetric-symmetric cell division through the transcriptional repression of the polarity protein LLGL1/2 by binding to their specific promoter regions, resulting in induced EMT and regulating the polarity of stem cell division to maintain the mammary epithelial homeostasis [70]. In addition to normal mammary epithelia, the epithelial polarity of breast cancer cells can be partially restored by knockdown of ZEB1 to accumulate LLGL2 in the cytoplasm [71].

### Tumor growth, metastasis, and EMT

**Fig. 2:**
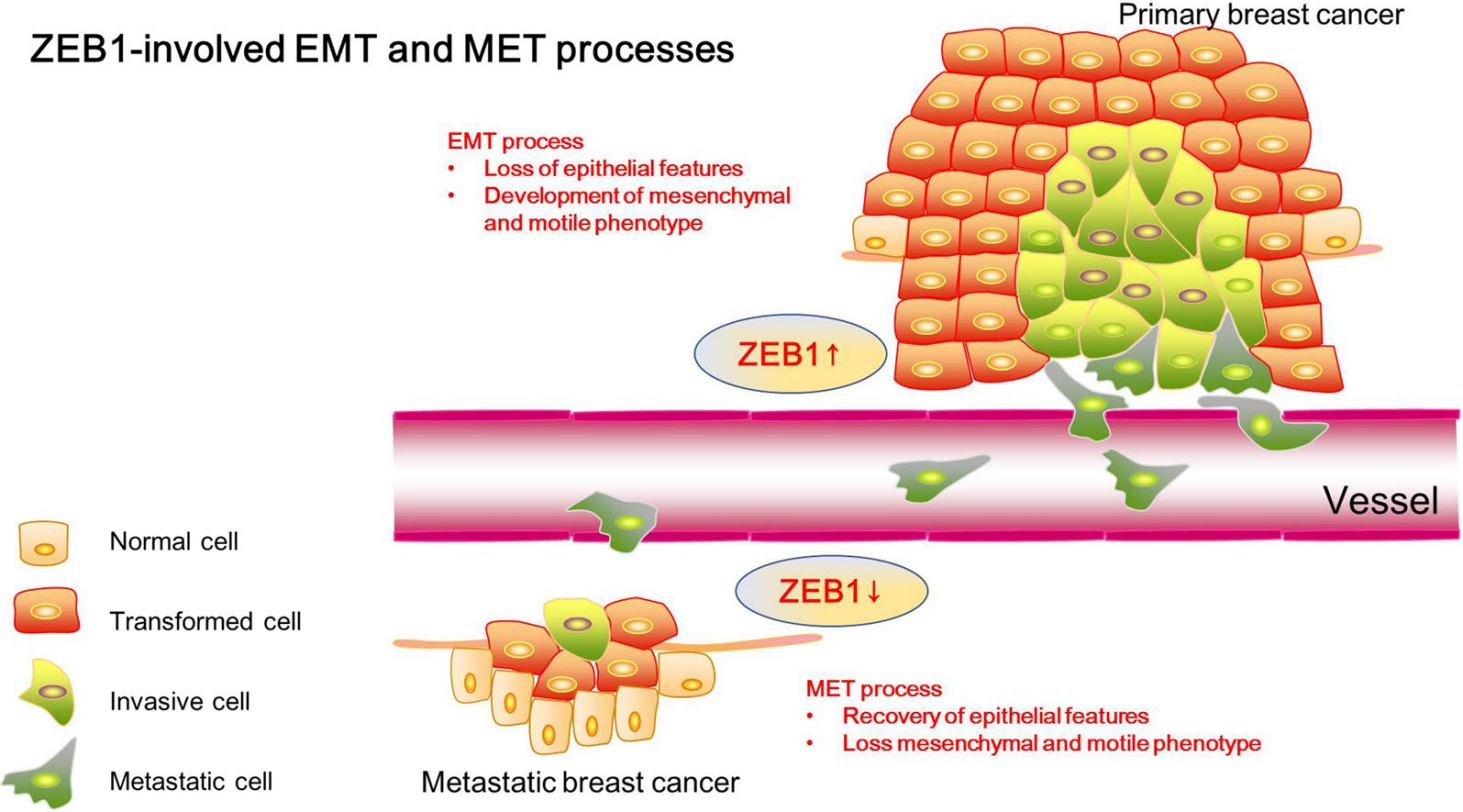
ZEB1-involved EMT and mesenchymal-epithelial transition (MET) processes. In primary breast cancer, the increased ZEB1 level suppressed the expression of the epithelial marker E-cadherin and induced the EMT process. The transformed tumor cells with high ZEB1 levels lost their epithelial characteristics, developed a mesenchymal/motile phenotype, and subsequently invaded into lymph or blood vessels. When metastatic breast cancer was formed in a distant location, the ZEB1 expression level was decreased to promote the expression of the epithelial marker E-cadherin and inhibit the expression of the mesenchymal marker Vimentin, occurring the mesenchymal-epithelial transition (MET) process to recover the epithelial features and lose the mesenchymal/motile phenotype with a low ZEB1 level

As shown in Fig. 2, the aberrant expression of ZEB1 is thought to be connected with tumorigenesis and poor prognosis in various tumors, especially in breast cancer [72–76]. In primary breast cancer, the increased ZEB1 suppressed the expression of epithelial marker E-cadherin and induced the EMT process, indicating that the transformed tumor cells with high ZEB1 level lost their epithelial characteristics and developed a mesenchymal/motile phenotype. Conversely, mesenchymal-epithelial transition (MET) process was occurred with decreased ZEB1 level, when metastatic breast cancer was formed in a distant location, to recover the epithelial features and lose the mesenchymal/motile phenotype with a low ZEB1 level (Fig. 2). Enhanced metastatic potential was associated with overexpression of ZEB1 in a mouse xenograft model of breast cancer, suggesting the role of ZEB1 in invasion and metastasis of human tumors [77]. On the other hand, ZEB1 contributes to the formation of the tumor microenvironment by regulating the levels of various inflammatory cytokines, such as interleukin 6/8 (IL-6/8), which resulted in increased tumor growth in basal-like breast cancer cells [78]. Recently, Fu et al. revealed the important role of the ZEB1/p53 axis in stromal fibroblasts to promote mammary epithelial tumors, which demonstrated the high expression and activation of ZEB1 in the stroma was associated with increased ECM remodeling, immune cell infiltration and angiogenesis through increasing FGF2/7, VEGF and IL-6 expression and secretion into the surrounding stroma [79].

Tumor vascularization predicted the deprivation of nutrients and oxygen and facilitated the metastasis process of tumor cells through vessel formation with or without endothelial transdifferentiation. During this process, the secreted proteins fibronectin 1 (FN1) and serine protease inhibitor family E member 2 (SERPINE2) are essential for vascular mimicry (VM) in this system. These secreted factors were upregulated in mesenchymal cells by ZEB1-repressed miRNA clusters, promoting autocrine signaling, which was followed by increased VM in breast cancer cells [80]. In clinical studies, ZEB1 was found to be significantly associated with the depth of invasion, lymph node metastasis and TNM stage in digestive cancer patients, as well as in patients with breast cancer [81].

### Chemoresistance

Currently, chemoresistance has become a major challenge and research hotspot in cancer treatment, referring to the increased tolerance of tumor cells to chemotherapeutic drugs and a decreased chemotherapeutic effect [82, 83]. CSCs have a self-renewal ability, can activate survival signaling pathways and can protect tumor cells from DNA damage and reactive oxygen species (ROS), contributing to drug resistance [84].

Increasing evidence indicates that ZEB1 has important significance in therapeutic resistance [85, 86]. Wang et al. found that breast cancer patients with high ZEB1 levels showed poor responses to epirubicin (EPI), indicating ZEB1 as the determinant of chemoresistance in breast cancer, involving DNA damage repair (DDR) [17]. In a large cohort of human breast cancer subjects, high levels of ZEB1 were shown to have positive relationships with Bcl-xl and cyclin D1, predicting a poor response to chemotherapy [77]. The researchers investigated the molecular mechanisms and found that the ZEB1/p300/PCAF (P300/CBP-associated factor, PCAF) complex bound to the ATM promoter and accordingly transcriptionally activated ATM, subsequently promoting homologous recombination (HR)-mediated DDR to clean up the chemotherapy-induced DNA fragments [77].

Endocrine therapy is an important therapeutic strategy for breast cancer patients with ER-positive expression; however, antiestrogen resistance has become a major obstacle in endocrine therapy and involves reduced estrogen receptor-alpha (ER-α) expression. Zhang et al. discovered that ZEB1 mechanistically inhibited ER-α transcription through the ZEB1/DNA methyltransferase 3B (DNMT3B)/histone deacetylase 1 (HDAC1) complex binding to hypermethylate and silence the ER-α promoter, subsequently attenuating the responsiveness of breast cancer cells to antiestrogen treatment [87]; these results indicate that ZEB1 is a key determinant of antiestrogen resistance in breast cancer.

Trastuzumab, a humanized monoclonal antibody against human epidermal growth factor receptor 2 (HER2), provides a successful therapeutic strategy for HER2-overexpressed breast cancer. Unfortunately, decreased trastuzumab sensitivities developed in most patients within a year [88]. Bai et al. noted that miR-200c/ZNF217/TGF-β/ZEB1, an expansile regulatory loop of the miR-200c/ZEB1 negative feedback circuit, synergistically increased trastuzumab sensitivity and suppressed the invasive abilities of breast cancer cells [50]. In this circuit, miR-200c suppressed the transcription factor ZNF217, which can promote the autocrine process of TGF-β; correspondingly, TGF-β signaling further transcriptionally activated ZEB1 to exhibit feedback suppression on the expression of miR-200c [49, 89].

β-catenin/TCF4 was also involved in chemoresistance through activating the transcriptional activities of ZEB1 [14, 60]. The nuclear accumulation of β-catenin was induced by Axl-activated Akt/GSK3β/β-catenin signaling, followed by a direct transcriptional increase in ZEB1, which in turn mediated DDR and doxorubicin resistance in breast cancer cells; these results suggest that the important function of Akt/GSK3β/β-catenin/ZEB1 signaling is downstream of Axl-mediated drug resistance [60].

### Radiation resistance

Radiotherapy is one of the major modalities of cancer treatment. A main reason for the failure of radiation therapy is intrinsic and therapy-induced radioresistant tumor cells, which display an enhanced DNA repair ability [90]. Although rare studies demonstrated the relationship between ZEB1 and radioresistance, the results still promoted corresponding scientific ideas to settle the problems caused by radiation resistance.

ZEB1 was identified to be phosphorylated and stabilized by ATM after ionizing radiation treatment in breast cancer cells, and correspondingly, the upregulation of ZEB1 was proven to stabilize checkpoint kinase 1 (CHK1) by activating the deubiquitylation of ubiquitin-specific-processing protease 7 (USP7), thus promoting homologous recombination-dependent DNA repair and resulting in radioresistance [43]. This study provided a potential mechanism for ZEB1 function in radioresistance, that is, ZEB1 was phosphorylated and stabilized by ATM depending on irradiation, which in turn repressed its negative regulator, miR-205, resulting in a further increase in ZEB1; these effects were followed by increased ubiquitin-conjugating enzyme Ubc13 levels and improved DDR, finally inducing radioresistance. Moreover, DDR was inhibited by miR-205 via targeting ZEB1 and Ubc13. Not surprisingly, targeting-ZEB1 agents, such as the miR-205 mimics, were supposed to be potential radiosensitizing agents, revealing a new therapeutic strategy for radioresistant tumors [91].

On the other hand, as ZEB1 was identified as a downstream target of miR-205, the expression level of miR-205 can be inhibited by nuclear enriched abundant transcript 1 (NEAT1), which regulates EMT progress and radioresistance in nasopharyngeal carcinoma [92]. Concerning the response of identified cancer cells to radiation, it is suggested that ZEB1 may both hinder the formation of oncogene-induced DNA damage by inhibiting oxidative stress and promote the clearance of DNA breaks by activating DDR, protecting cancer cells for survival [16].

## Conclusion

**Fig. 3:**
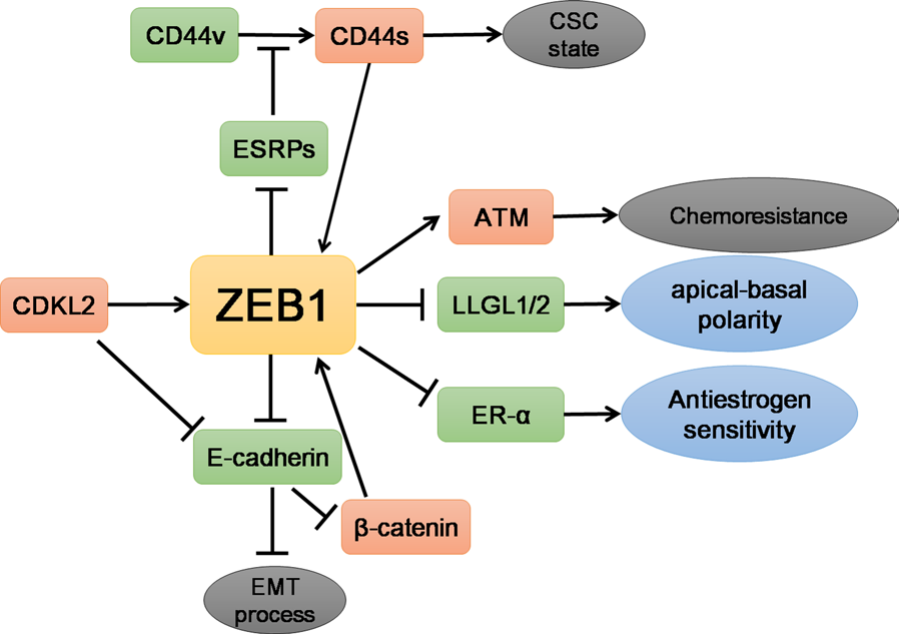
The main targets of ZEB1 and the processes involved in breast cancers. Cyclin-dependent protein kinase-like 2(CDKL2) transcriptionally activates ZEB1 and suppresses E-cadherin to promote the EMT process. The carcinogenesis-related CSC state is determined by the CD44s isoform conserved by CD44v and controlled by epithelial splicing regulatory protein 1 (ESRP1). The transcriptional suppression of downstream LLGL1/2 by ZEB1 is associated with apical-basal polarity, and inhibition of ER-α by ZEB1 regulates the sensitivities to antiestrogen treatment in breast cancer. However, ZEB1 also transcriptionally activates the expression of ATM kinase and promotes the occurrence of chemoresistance

In recent decades, the understanding of the molecular mechanism of ZEB1 in cancer progression has greatly improved (Fig. 3). Accumulated evidence indicates that ZEB1 displays a broad spectrum of biological functions. Elevated expression of ZEB1 not only increases cell motility and invasiveness by downregulating epithelial markers and upregulating mesenchymal markers but also contributes stem cell-like features to tumor cells, providing resistance to various types of therapy. Therefore, the expression of ZEB1 may be a biomarker of poor clinical outcomes for cancer patients. The recent recognition of the regulation and functions of ZEB1 has shed new light on understanding its potential clinical and therapeutic implications in cancers.

Despite these developments, much remains unknown about the role of ZEB1 in metastasis, a complex and multistep process where cancer cells hijack the normal developmental networks for tumor progression and metastasis. Within the complex signaling networks, ZEB1 is regulated through signal integration, crosstalk and feedback control. It is often difficult to identify whether a particular molecule or pathway under investigation is specific to ZEB1-mediated EMT. Therefore, further investigations to reveal the contribution of various tumor microenvironmental factors to tumor progression will lead to a comprehensive understanding of ZEB1 in cancer.

Recent studies have shown that the suppression of the miR-200 family by ZEB1 results in the upregulation of programmed cell death protein 1 (PD-L1), which is related to the immune system [93], suggesting that the ZEB1/miR-200 axis could influence the immune recognition of cancer cells. Therefore, in addition to normal treatments for cancers, the exploration of ZEB1 in immunotherapy has provided new potential and effective therapeutic strategies for metastatic breast cancers.

## Data Availability

Not applicable.

## Abbreviations

ATM: Ataxia-telangiectasia mutated kinase
AXL: AXL receptor tyrosine kinase
BET: Bromodomain and extra-terminal
bHLH: Basic helix-loop-helix
CBS: Cascading bistable switches
CDKL2: Cyclin-dependent protein kinase-like 2
CHK1: Checkpoint kinase 1
CSC: Cancer stem cells
CTGF: Connective tissue growth factor
CZF: C-terminal cluster
DDR: DNA damage repair
DNMT3B: DNA methyltransferase 3B
EMT: Epithelial-mesenchymal transition
EPI: Epirubicin
ER-α: Estrogen receptor-alpha
ESRPs: Epithelial splicing regulatory proteins
HD: Homeodomain
HDAC1: Histone deacetylase 1
HER2: Human epidermal growth factor receptor 2
HNK: Honokiol
HR: Homologous recombination
IGF1: Insulin-like growth factor-1
IL: Interleukin
LLGL1/2: Lethal giant larvae homolog 1/2
NEAT1: Nuclear enriched abundant transcript 1
NZF: N-terminal cluster
PCAF: P300/CBP-associated factor
PD-L1: Programmed cell death protein 1
PROTAC: Proteolysis targeting chimeric
ROS: Reactive oxygen species
STAT: Signal transducer and activator of transcription
SWI/SNF: Yeast mating-type switching/sucrose nonfermenting
TCS: Ternary chimera switch
TGF: Transforming growth factor
TNBC: Triple-negative breast cancers
TNF: Tumor necrosis factor
USP7: Ubiquitin-specific-processing protease 7
UTR: Untranslated region
VM: Vascular mimicry
YAP: Yes-associated protein
ZEB: Zinc finger E-box binding homeobox
ZNF: Zinc finger protein
